# Decreased Survival With Mastectomy Vis-à-Vis Breast-Conserving Surgery in Stage II and III Breast Cancers: A Comparative Treatment Effectiveness Study

**DOI:** 10.1200/JGO.2016.004614

**Published:** 2016-10-12

**Authors:** Ambakumar Nandakumar, Goura Kishor Rath, Amal Chandra Kataki, P. Poonamalle Bapsy, Prakash C. Gupta, Paleth Gangadharan, Ramesh C. Mahajan, Manas Nath Bandyopadhyay, Kumara Swamy, Elizabeth Vallikad, Rudrapatna N. Visweswara, Francis Selvaraj Roselind, Krishnan Sathishkumar, Dampilla Daniel Vijay Kumar, Ankush Jain, Kondalli Lakshminarayana Sudarshan

**Affiliations:** **Ambakumar Nandakumar**, **Francis Selvaraj Roselind**, **Krishnan Sathishkumar**, **Dampilla Daniel Vijay Kumar**, **Ankush Jain**, **Kondalli Lakshminarayana Sudarshan**, National Centre for Disease Informatics and Research; **P. Poonamalle Bapsy**, Apollo Hospitals; **Kumara Swamy**, HealthCare Global–Bangalore Institute of Oncology; **Elizabeth Vallikad**, St John’s Medical College; **Rudrapatna N. Visweswara**, International Medical School–MS Ramaiah Medical College, Bangalore; **Goura Kishor Rath**, Institute Rotary Cancer Hospital, All India Institute of Medical Sciences, New Delhi; **Amal Chandra Kataki**, Dr B.B. Borooah Cancer Institute, Guwahati; **Prakash C. Gupta**, Healis-Sekhsaria Institute of Public Health, Navi Mumbai; **Paleth Gangadharan**, Amrita Institute of Medical Sciences and Research Centre, Kochi; **Ramesh C. Mahajan**, Postgraduate Institute of Medical Education and Research, Chandigarh; and **Manas Nath Bandyopadhyay**, Cancer Centre Welfare Home and Research Institute, Kolkata, India.

## Abstract

**Purpose:**

The primary purpose of hospital-based cancer registries is assessing patient care. Clinical stage–based survival and treatment-based survival are some of the key parameters for such assessment. Because of the challenges in obtaining follow-up parameters, a separate study on patterns of care and survival was undertaken by the Indian National Cancer Registry Program. The results for cancer of the female breast are presented here.

**Patients and Methods:**

Data abstracted in a standardized patient information form were transmitted online to a central repository. Treatment patterns were assessed for 9,903 patients diagnosed between January 1, 2006, and December 31, 2008, from 13 institutions. Survival analysis was restricted to 7,609 patients from nine institutions wherein follow-up details (as of December 31, 2012) were available for at least 60% of patients.

**Results:**

The overall 5-year survival rates with breast-conserving surgery (BCS) and mastectomy (MS) were 94.0% and 85.8%, respectively, for stage II disease (adjusted hazard ratio, 2.40; 95% CI, 1.8 to 3.2) and 87.1% and 69.0%, respectively, for stage III disease (hazard ratio, 2.82; 95% CI, 2.2 to 3.7). Patients who had MS did better with systemic therapy (chemotherapy and/or hormone therapy), whereas patients with BCS required just local radiation therapy to achieve best survival.

**Conclusion:**

This observational study in the natural setting of care of patients with cancer in India showed significantly decreased survival with MS when compared with BCS. The reasons for lower survival with MS and the biologic or scientific rationale of the necessity of systemic therapy to achieve optimal survival in patients undergoing MS but not in those with BCS need further investigation.

## INTRODUCTION

Worldwide, breast cancer is the most common cancer among women, composing 25% of all female cancers.^1^ The annual estimated incidence of new breast cancers in women in India is approximately 100,000. Breast cancer has shown a statistically significant increase in incidence rates over time in the Indian Population-Based Cancer Registries.^2^ Information on patterns of breast cancer care and survival is essential in assessing cancer treatment services, and a hospital-based cancer registry is central to this effort.^3^ There are several challenges in obtaining proper and accurate data on clinical stage–based survival in the setting of a developing country. These include treatment compliance and post-treatment follow-up, including relevant information on recurrence and/or complications of disease.^4^ The main aim of this study was to obtain clinical stage–specific treatment and survival information for breast cancer in India.

Numerous studies from developed countries have compared survival results of breast-conserving surgery (BCS) and mastectomy (MS).^5-8^ Previous publications from India on BCS and survival are from individual hospitals.^9-11^ The findings pooled and reported in this study were facilitated by a process of electronic transfer of data from collaborating institutions with the Internet as the medium of transmission to a central repository. An earlier version of this method, which constituted the basic design and framework for obtaining information, has been described previously.^12^

## PATIENTS AND METHODS

Thirteen institutions participated in the study (Data Supplement). A standardized patient information form (PIF; Data Supplement) developed by oncologists with specific expertise in breast cancer was hosted on a Web site (www.hbccrindia.org). Printed forms with an instruction manual were supplied to participants. Trained staff completed the form through a combination of patient-attendant interviews; scrutiny of medical records, other relevant documents, and registers; and discussions with concerned clinicians. Collaborating centers were given individual login identifications and passwords with instructions for online data entry to electronically transmit the data to a central repository, the National Centre for Disease Informatics and Research (NCDIR). The mandate and mission statements of this unique center (an outcome of the National Cancer Registry Program of the Indian Council of Medical Research) are provided at the NCDIR Web site (www.ncdirindia.org). All participating institutions had the study protocol cleared by their respective institutional ethics committees, and patient consent was incorporated into the individual patient medical record.

Stage-based treatment patterns were examined for 9,903 newly diagnosed (between January 1, 2006, and December 31, 2008) patients with infiltrating duct carcinoma of the breast treated at the participating institutions. However, survival analysis was restricted to 7,609 patients from nine centers having at least 60% follow-up information on their respective patients as of December 31, 2012. The ultimate end point of overall survival (OS) was defined as the period between the date of diagnosis and the date of death (when death occurred before January 1, 2013) from any cause. Patients who died on or after January 1, 2013, were deemed alive for survival analysis. The number and proportion of patients with toxicity (on the basis of early and late complications) and recurrence are based on any one such reported event. The PIF provided details of clinical TNM and stage grouping at initial presentation of the patient. On the basis of the lymph node status, pathologic TNM (pTNM) was also recorded. TNM on the basis of histopathology findings (pTNM) superseded the clinical TNM for the final stage grouping.^13^ Analysis was carried out separately for stages I, II, and III. Patients with stage IV disease (which is exclusively a metastatic disease) and patients whose stage was unknown were excluded from this study.

### Surgical Treatment

Patients who had undergone lumpectomy with or without axillary lymph node clearance were defined as having received BCS, whereas patients who had simple MS (with or without axillary lymph node clearance) or radical MS were classified as having had MS.

### Radiotherapy

Patients who had received radiotherapy (RT) to the chest wall (≥ 45 Gy) with or without an additional boost with radical intent were considered as having received optimal RT, and those who received less than this dose were considered as have received suboptimal RT. Patients treated with palliative intent RT or RT given only to the axilla and/or supraclavicular nodes were excluded. Few patients (1.6%) received RT through such techniques as intensity-modulated RT or image-guided RT, so these factors were disregarded.

### Chemotherapy

Chemotherapy was considered as given if the patient received it as neoadjuvant, concurrent, or adjuvant chemotherapy. Standard prescribed protocols in use of specific chemotherapy drugs were followed. Anthracyclines with and without taxanes and/or other drug combinations were analyzed separately. Because few patients (n = 23) were given trastuzumab, no separate analysis was done on this.

### Hormone Therapy

Patients who received neoadjuvant or adjuvant intervention with surgical oophorectomy, RT-ovarian ablation, medical tamoxifen, or aromatase inhibitors were considered as having received hormone therapy (HT).

### Receptor Status

Estrogen receptor (ER), progesterone receptor (PR), and human epidermal growth factor receptor 2 (HER2) status was determined in all patients and was classified as triple negative, triple positive, ER and PR positive and HER2 negative, and other combinations.

### Software Programs and Quality Checks

In-house Internet-based software programs (www.hbccrindia.org; www.ncdirindia.org) were modeled for data capture, checked for completeness and consistency, tracked patient follow-up, updated treatment information, and recorded follow-up details. Checks done on data varied from date checks to verifying discrepancies in clinical information (Data Supplement). Lists of patients with improbable data were sent back to concerned centers for rectification. Further, a center-wise random sample of 10% of patients was listed, and centers were asked to reabstract the medical records for certain essential parameters; the matching results were found to be 95% accurate.

### Statistical Analysis

The Kaplan-Meier method^14^ and Cox proportional hazards regression^15^ in the SPSS package (version 21; SPSS, Chicago, IL) were used to calculate 5-year cumulative survival (FCS) rate and fatality risk (with statistical significance), respectively. Multivariate analysis was performed using Cox proportional hazard regression analysis.

## RESULTS

The Data Supplement provides patient, diagnostic, and treatment characteristics for the 9,903 patients with breast cancer in whom patterns of care were examined and for the 7,609 patients in whom survival analysis was also done. Overall, the FCS rate in 7,609 patients was 73.8%, varying from 93.3% in patients with stage I disease to 24.5% in patients with stage IV disease.

### Stage I

BCS was performed in 198 (55.9%) of 354 patients, and MS was performed in 146 (41.2%) of 354 patients. The median ages of women who had BCS and MS were 52 and 57 years, respectively. The FCS rates were 95.5% for patients who had BCS (alone or with other treatments) and 91.3% for patients who underwent MS (alone or with other treatments). The difference was not statistically significant (hazard ratio [HR], 1.7; 95% CI, 0.7 to 4.2). Patients who received RT or HT in addition to surgery (BCS or MS) had significantly better survival compared with those who did not receive RT or HT (RT: FCS, 97.6% *v* 88.8%, respectively; HR, 4.0; 95% CI, 1.5 to 10.9; HT: FCS, 96.7% *v* 88.3%, respectively; HR, 3.0; 95% CI, 1.3 to 7.1). A combination of surgery (BCS or MS), RT, and HT with or without chemotherapy gave significantly better OS compared with all other single or multimodality treatments (FCS, 99.0% *v* 90.7%, respectively; HR, 5.1; 95% CI, 1.2 to 21.7). Survival in stage I breast cancers was not influenced by age or receptor status.

### Stage II

Of 2,880 patients with stage II disease, BCS was performed in 965 patients (33.5%), MS was performed in 1,785 patients (62.0%), and 130 patients (4.5%) did not receive any type of surgery. The patient, diagnostic, and treatment characteristics of patients who received BCS and MS are given in the Data Supplement. Patients who had BCS, compared with those who had MS, showed significantly better survival (FCS, 94.0% *v* 85.8%, respectively; Table 1). Patients with MS also had a higher risk of death after adjusting for all other types of treatments and receptor status (HR, 2.4; 95% CI, 1.7 to 3.3). Survival was significantly better in patients who had ER- and PR-positive and HER2-negative disease than in patients with triple-negative receptor status.

**Table 1:**
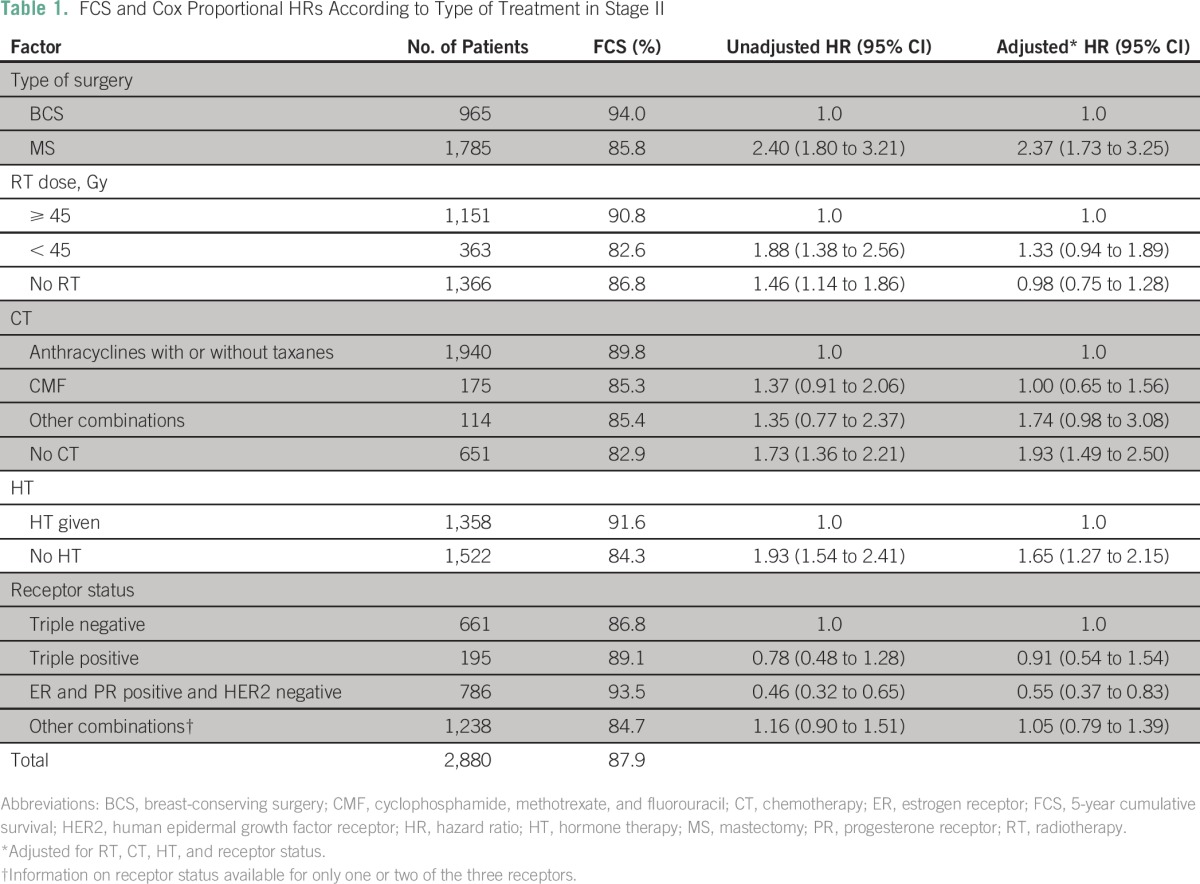
FCS and Cox Proportional HRs According to Type of Treatment in Stage II

Analysis was done separately for the BCS and MS groups to examine the possible reasons for the relatively poorer survival in patients with MS (Tables 2 and 3). For patients with BCS, inclusion of RT, which constitutes breast-conservation therapy (BCT), significantly increased the survival compared with patients who had not received RT. For patients who had MS, addition of both chemotherapy and HT significantly increased survival, whereas there was no improvement in survival with addition of RT (Table 3).

**Table 2:**
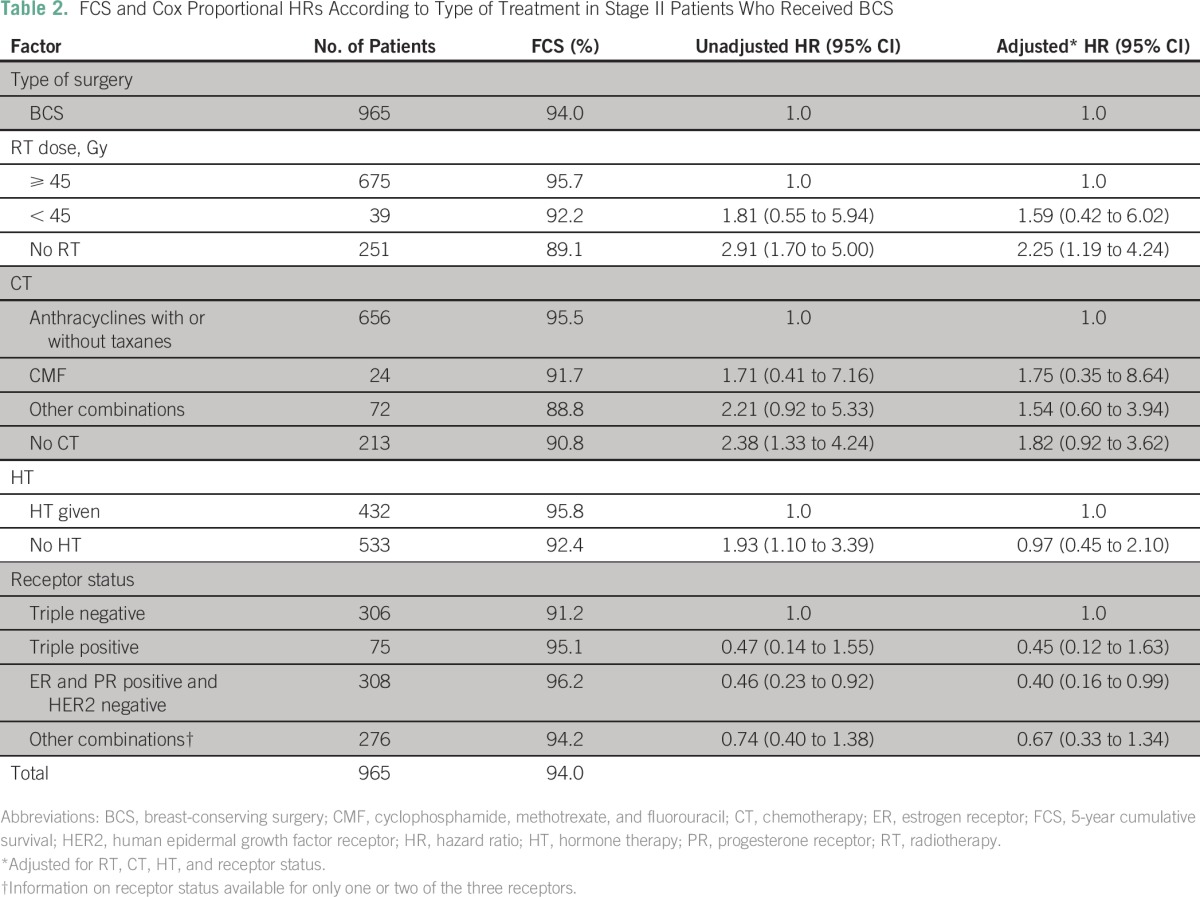
FCS and Cox Proportional HRs According to Type of Treatment in Stage II Patients Who Received BCS

**Table 3:**
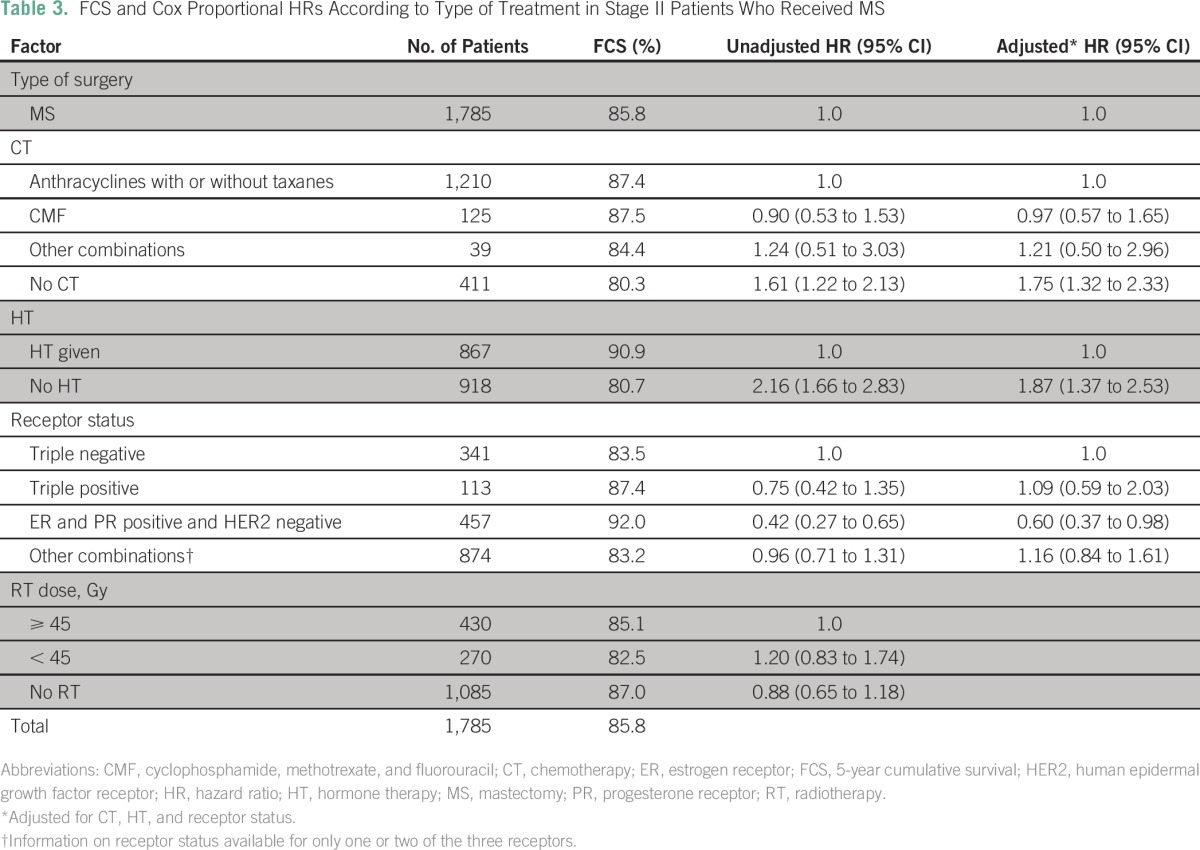
FCS and Cox Proportional HRs According to Type of Treatment in Stage II Patients Who Received MS

The overall findings were similar when analysis was done separately for stage IIA and IIB disease. However, when examined by node status (N0 [node negative] and N1 [node positive]), the difference in the FCS rate between BCS and MS was more pronounced in node-positive patients (11.6% [92.8% *v* 81.2%, respectively]) compared with node-negative patients (5.0% [95.0% *v* 90.0%, respectively]). Furthermore, node-positive MS patients showed significantly poorer survival in the absence of administering optimal RT (HR, 2.7; 95% CI, 1.5 to 4.9), chemotherapy (HR, 1.97; 95% CI, 1.2 to 3.2), or HT (HR, 2.1; 95% CI, 1.3 to 3.4) compared with MS patients who had received optimal RT, chemotherapy, or HT.

### Stage III

Of the 3,620 patients with stage III disease, 544 (15.0%) underwent BCS, and 2,740 (75.7%) underwent MS. Three hundred thirty-six patients (9.3%) did not receive any surgical treatment. The patient, diagnostic, and treatment characteristics of patients who received BCS and MS are given in the Data Supplement. Table 4 lists the FCS rates and Cox proportional hazards HRs according to type of treatment. The FCS rate was 87.1% for BCS compared with 69.0% for MS. The risk of death for MS compared with BCS as expressed by the HR was significantly higher (HR, 2.6; 95% CI, 2.0 to 3.4) after adjusting for RT, chemotherapy, and HT. One hundred ninety-four patients received cyclophosphamide, methotrexate, and fluorouracil as chemotherapy and had significantly poorer survival compared with patients who received anthracyclines with or without taxanes as chemotherapy.

**Table 4:**
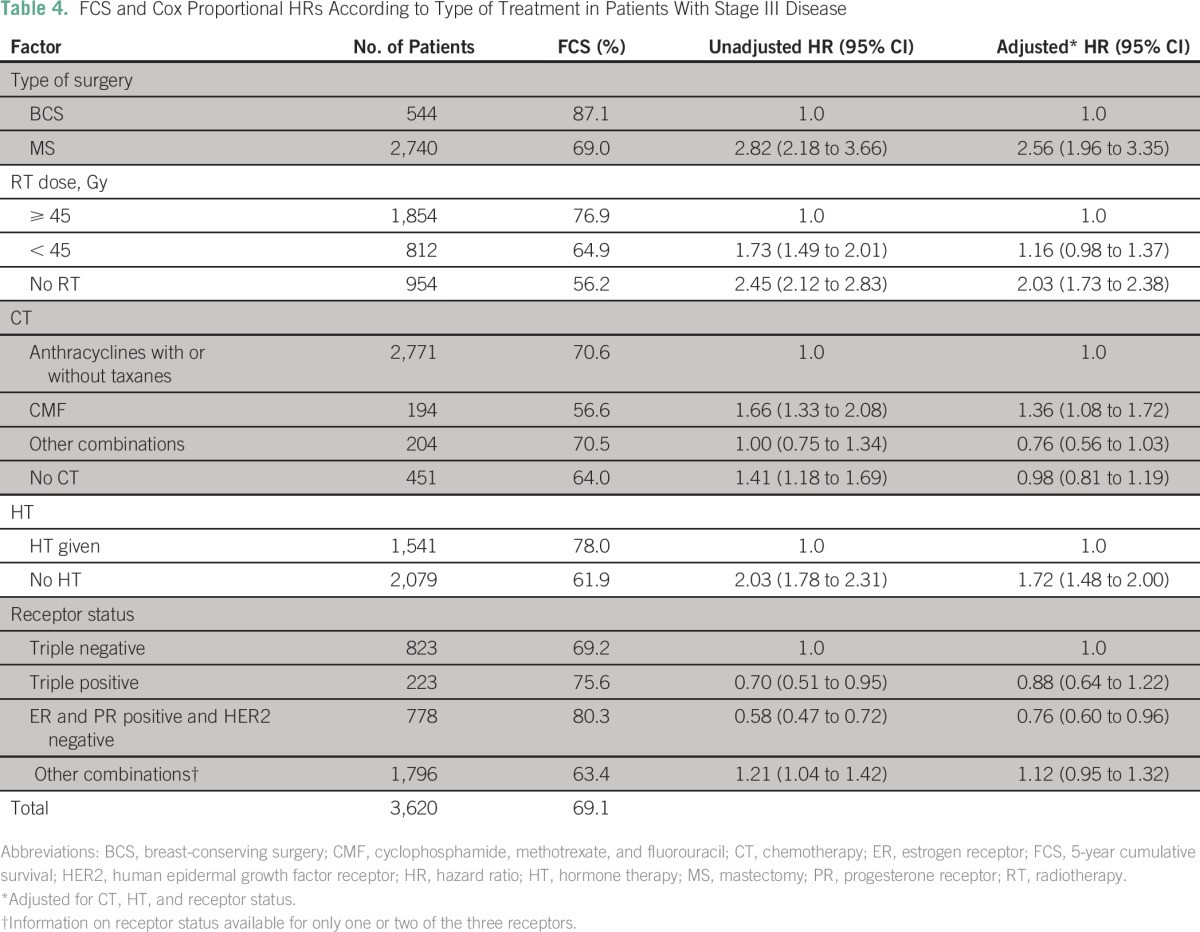
FCS and Cox Proportional HRs According to Type of Treatment in Patients With Stage III Disease

As in patients with stage II disease, the overall findings were similar when analysis was done separately for stage IIIA and stage IIIB. Also as in patient with stage II disease, node-positive MS patients (N1-3) had a significantly lower survival when optimal RT and/or HT were not administered compared with MS patients who had received these therapies.

Table 5 compares the survival rates in this study with those in other key reports. The survival benefit of BCS, especially in stage II and III breast cancer, seems to be greater in this study when compared with others. The Data Supplement depicts the Kaplan-Meier comparative survival curves for patients who received BCS and MS separately for stage II and stage III disease. There was no survival difference between age groups in patients with stage II or stage III disease.

**Table 5:**
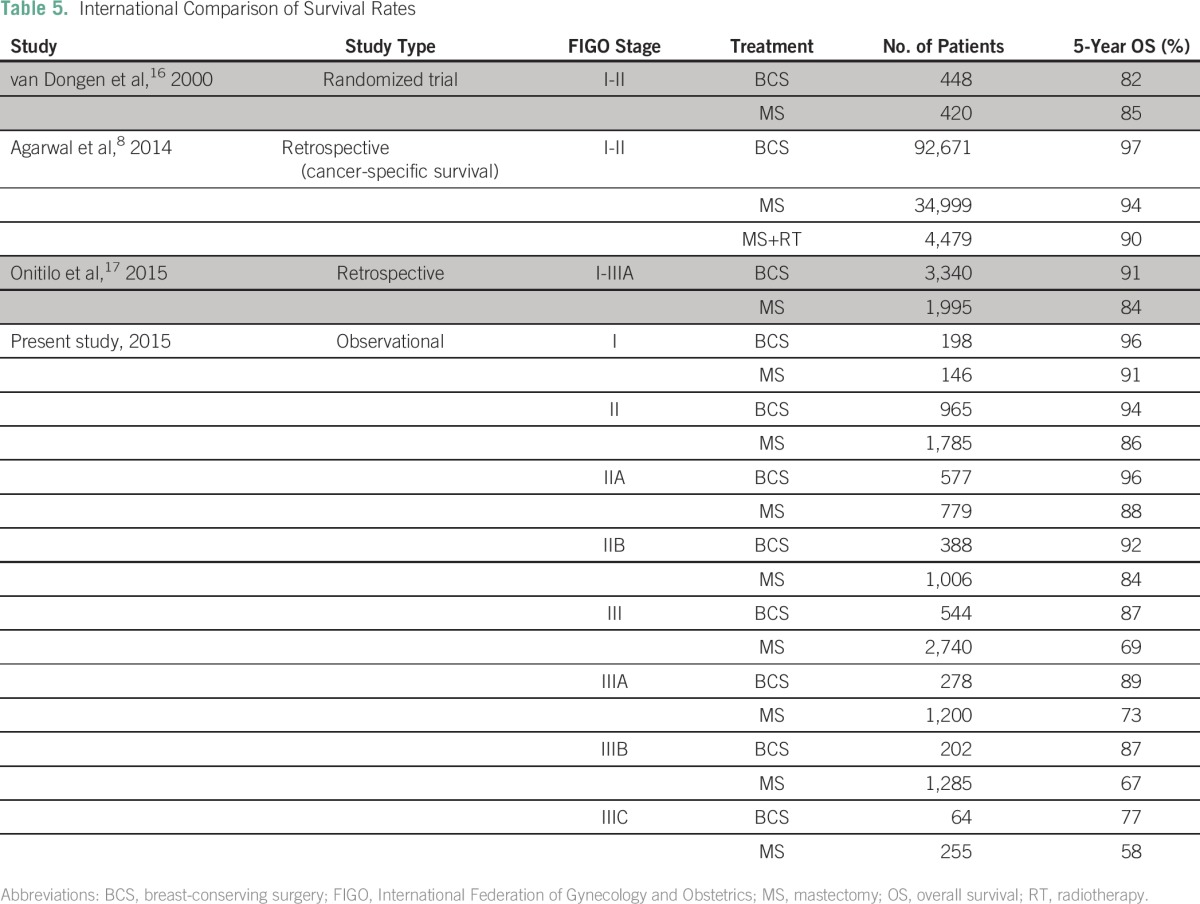
International Comparison of Survival Rates

## DISCUSSION

Several clinical trials have shown that BCS is as good as MS in early-stage (stage II) breast cancer, although some reports have indicated a higher rate of local recurrence in patients who have undergone BCS.^5,16,18,19^ Other aspects that have been compared are initial tumor size, patterns of recurrence, and time to locoregional recurrence or distant metastases.^6,20^ These studies have shown that there is no difference in survival between the two types of surgery. A national registry-based stage-by-stage comparison has also shown equal survival for BCS and MS.^21^ A meta-analysis by Yang et al^22^ showed that OS was not statistically significant between patients who underwent MS and BCT.

However, recent publications from Canada^23^ and results from the SEER database^8^ show better survival of patients with BCT compared with MS. A report presented at the San Antonio Breast Cancer Symposium in December 2015 on an observational study of 37,000 women showed a better survival with BCT than with MS (76.8% *v* 59.7%, respectively).^24^

There have been single-institution reports from India comparing survival in patients receiving BCS and those receiving MS.^10,11^ To our knowledge, this is the first multi-institutional study in India to provide insight into the extent of practice of BCS vis-à-vis MS and the survival differences between these two treatments. It is not a randomized clinical trial, but it nonetheless gives an accurate picture of the patterns of care and survival in the natural arena of selection of patients with cancer for a specific type cancer treatment in the country. Observational studies such as the patterns of care studies or comparative treatment effectiveness studies have the following several advantages: they are comprehensive, have been externally validated, have broader criteria for patient inclusion, are unbiased, and provide results in routine clinical settings.^25^ The strengths, opportunities, and limitations of this study, which is identical to the one on cervical cancer, have been outlined previously.^26^ The differences observed in survival are a result of a combination of treatment modality and selection criteria of patients. The latter are unknown and have not been taken into account. However, there is no selection bias because all patients who received cancer-directed treatment in their respective institutions have been accounted for, and exclusion criteria are based on scientific logic.^26^ Other limitations specific to this study are that in a high proportion of patients (pattern of care, 33.4%; pattern of survival, 28.6%) information on receptor status was not available and a substantial number of patients with stage I (10 patients), stage II (130 patients), and stage III disease (336 patients) did not receive any surgical treatment. Because there was no information as to why surgery was not done in these patients, we were unable to provide the clinical or other rationale for this result.

This study is a foremost example of cancer registration (through a national program of cancer registries covering several cancer centers and medical institutions) evaluating clinical parameters and providing critical findings that could have an impact on patient care. A network of cancer hospitals linked to a central coordinating center (NCDIR) with a system to accrue good clinical data through modern electronic information technology is in place.

The differences in survival that were observed between MS and BCS are significant and substantial. The difference was 8.2% for patients with stage II disease and as high as 18.1% for patients with stage III disease, with the results being the same even when separately analyzed according to stages IIIA and IIIB. The results presented here are those of pooled data from several institutions. The data of individual centers were separately examined to ascertain whether there were any variations among institutions in the survival or the extent of survival difference between BCS and MS. A comparable survival pattern and difference were observed in all institutions. Age and rate of recurrence or complications were also similar between the two groups. The variation in other treatments impacting survival between patients who had received BCS and MS shows that for stage II disease, RT plays a major role for BCS but not for MS, although relatively more MS patients received less RT and suboptimal RT. The roles of chemotherapy and HT seem less important than RT for BCS, whereas these treatments (chemotherapy and HT) are more important for improved survival in the MS group. The need for greater systemic therapy in patients with MS in contrast to those who had BCS is difficult to explain. One could hypothesize that removal of the breast as a result of MS entails loss of patient immunity that requires additional systemic therapy.

A similar picture was observed in stage III disease where patients who had MS did better when they received a combination of RT, chemotherapy, and HT, whereas in patients who had BCS, RT alone was sufficient to have equivalent survival. The proportions of patients with stage II and III disease in the pattern-of-care group (all 13 institutions) who underwent BCS were 29.5% and 12.8%, respectively (Data Supplement).

In conclucion, the findings justify further investigation of the possible reasons for decreased survival in patients with MS. The underlying cause of death, including the events that lead to it, whether a result of early or late complications of treatment, needs to be studied. A well-substantiated analysis of this parameter would require availability of accurate and complete information on these characteristics in a majority of patients, which was not available in the current study. Because obtaining such data is hugely challenging in the routine Indian setting, a randomized study on the cause of death in patients with breast cancer could provide insights. Such studies could take into account additional and further details of points that were not gathered in this study, including information on individual caregivers, socioeconomic status of patients, and additional details on investigations done for staging. There seems to be a need to promote awareness about the distinct advantages of BCS in the context of developing countries, and further studies are required to examine the biologic or scientific rationale of the requirement of systemic therapy in patients with MS but not in those with BCS.

## Appendix

### Patterns of Cancer Care and Survival Studies Group

Project Management Group: Ambakumar Nandakumar (National Centre for Disease Informatics and Research [NCDIR], Bangalore), Francis Selvaraj Roselind (NCDIR), Krishnan Sathishkumar (NCDIR), Dampilla Daniel Vijay Kumar (NCDIR), Ankush Jain (NCDIR), and Kondalli Lakshminarayana Sudarshan (NCDIR).

Cancer Research Area Panel: Goura Kishor Rath (Institute Rotary Cancer Hospital, All India Institute of Medical Sciences, New Delhi), Amal Chandra Kataki (Dr B.B. Borooah Cancer Institute, Guwahati), P. Poonamalle Bapsy (Apollo Hospitals, Bangalore), Prakash C. Gupta (Healis-Sekhsaria Institute of Public Health, Navi Mumbai), Paleth Gangadharan (Amrita Institute of Medical Sciences and Research Centre, Kochi), Ramesh C. Mahajan (Postgraduate Institute of Medical Education and Research, Chandigarh), Manas Nath Bandyopadhyay (Cancer Centre Welfare Home and Research Institute, Kolkata), Kumara Swamy (HealthCare Global–Bangalore Institute of Oncology, Bangalore), Elizabeth Vallikad (St John’s Medical College, Bangalore), Rudrapatna N. Visweswara (International Medical School–MS Ramaiah Medical College, Bangalore).

Collaborators (institutes) supplying individual patient data (in descending order of the number of patients with cancer on whom information was provided, first for survival studies and then for pattern of care only): Tata Memorial Hospital (TMH), Mumbai—R.A. Badwe; A.K. D’Cruz; B. Ganesh; Vani Parmar; Sudeep Gupta; Ashwini Budrukkar; Rakesh Jalali; Arshi Khan; Sushama Saoba; Sharwari Joshi; Mitali Sapkal. Cancer Institute (WIA), Chennai—V. Shanta; R. Swaminathan; V. Sridevi; R. Rama; P. Shanthi; M.S. Kalyani. Regional Cancer Centre, Thiruvananthapuram—Paul Sebastian; Aleyamma Mathew; Preethi Sara George; Ratheesan K; Beela Sarah Mathew. Amrita Institute of Medical Sciences, Kochi—D.K. Vijaykumar; P. Gangadharan. Mahavir Cancer Sansthan, Patna—J.K. Singh; M. Singh; P. Jain; A. Kumari. HealthCare Global Bangalore Institute of Oncology, Bangalore—B.S. Ajaikumar, Kumaraswamy, Raghavendra Rao M, M.Bandemegal, K.Murugan, N.Rao, R.S. Bilimagga, Gopinath K. S, R. Nayak. Dr B. Borooah Cancer Institute, Regional Cancer Centre, Guwahati—A.C. Kataki; Mouchumee Bhattacharyya. Assam Medical College, Dibrugarh—K. Adhikari M; M.S. Ali; R. Akhtar; S.K. Bhuyan; I. Baruah; Sheila Neog. Cachar Cancer Hospital and Research Centre, Silchar—R. Kannan; R. Tapkire; G. Dutta; A. Das; G. Roy. Rajiv Gandhi Cancer Institute and Research Centre, New Delhi—S. Rawat; A. Mishra; A.K. Pahuja. Postgraduate Institute of Medical Education and Research, Chandigarh—S. Ghoshal; S.C. Sharma. Kidwai Memorial Institute of Oncology, Bangalore—M. Vijayakumar; K. Ramachandra Reddy; C. Ramesh; D.J. Jayaram. Government Medical College and Hospital, Nagpur—K.M. Kamble; V.N. Sagdeo; N. Jambhulkar. ICMR Headquarters, New Delhi—V.M. Katoch; D.K. Shukla; T. Kaur.
